# Prenatal Glucocorticoid Treatment and Later Mental Health in Children and Adolescents

**DOI:** 10.1371/journal.pone.0081394

**Published:** 2013-11-22

**Authors:** Natasha Khalife, Vivette Glover, Anja Taanila, Hanna Ebeling, Marjo-Riitta Järvelin, Alina Rodriguez

**Affiliations:** 1 Department of Epidemiology and Biostatistics, Imperial College London, London, United Kingdom; 2 Institute of Reproductive and Developmental Biology, Imperial College London, London, United Kingdom; 3 Institute of Health Sciences, University of Oulu, Oulu, Finland; 4 Unit of General Practice, Oulu University Hospital, Oulu, Finland; 5 Institute of Clinical Medicine, Clinic of Child Psychiatry, University of Oulu, Oulu, Finland; 6 Clinic of Child Psychiatry, Oulu University Hospital, Oulu, Finland; 7 Unit of Primary Care, Oulu University Hospital, Oulu, Finland; 8 MRC Health Protection Agency (HPA) Centre for Environment and Health, Imperial College London, London, United Kingdom; 9 Department of Children and Young People and Families, National Institute for Health and Welfare, Oulu, Finland; 10 Biocenter Oulu, University of Oulu, Oulu, Finland; 11 Department of Psychology, Mid Sweden University, Östersund, Sweden; The University of Queensland, Australia

## Abstract

**Background:**

Animal studies demonstrate a clear link between prenatal exposure to glucocorticoids (GC) and altered offspring brain development. We aim to examine whether prenatal GC exposure programs long-term mental health in humans.

**Methods:**

Using propensity-score-matching, children prenatally exposed to synthetic glucocorticoids (sGC), n=37, and controls, n=185, were balanced on important confounders related to sGC treatment - gestational age and pre-pregnancy BMI. We also used mixed-effects modeling to analyse the entire cohort – matching each sGC case, n=37, to all possible controls, n=6079, on gestational age and sex. We obtained data from the Northern Finland Birth Cohort 1986 at four waves – pregnancy, birth, 8 and 16 years. Data on pregnancy and birth outcomes came from medical records. Mental health was assessed at 8 years by teachers with the Rutter B2 scale, and at 16 years by parents with the Strengths and Weaknesses of ADHD symptoms and Normal behavior (SWAN) scale and adolescents by the Youth Self-Report (YSR) scale.

**Results:**

Prenatal sGC treatment was consistently associated with adverse mental health in childhood and adolescence, as shown by both the propensity-score method and mixed-effects model. Using the propensity-score-matched subsample, linear multiple regression showed prenatal sGC was significantly linked with general psychiatric disturbance (B=8.34 [95% CI: .23-16.45]) and inattention (B= .97 [95% CI: .16-1.80]) at 8 years after control for relevant confounders. Similar findings were obtained at 16 years, but did not reach statistical significance. Mediation by birthweight/placental weight was not detected.

**Conclusions:**

This study is the first to prospectively investigate the long-term associations between prenatal exposure to sGC treatment and mental health in children and adolescents. We report an association between prenatal exposure to sGC and child mental health, supportive of the idea that sGC has a programming effect on the fetal brain.

## Introduction

Cortisol, a naturally occurring glucocorticoid (GC), plays a vital role in fetal development [1]. This hormone exerts a wide range of effects in most regions of the developing brain, initiating terminal maturation, remodeling of axons and dendrites, and affecting cell survival [2]. However, sustained elevation or reduction of GC levels can impair these processes, and thereby permanently modify brain structure and function [3], suggesting a role for GC in fetal programming of mental health. Fetal exposure to elevated levels of maternal cortisol has been proposed as one mechanism underlying the reported connection between prenatal exposure to maternal stress and symptoms of attention-deficit/hyperactivity disorder (ADHD) in the offspring [4-6]. ADHD is the most common behavioral disorder in young people, characterized by inappropriate inattention, hyperactivity and impulsivity [7,8], and is related to impairments in all areas of life e.g. social and scholastic domains [9]. 

Animal models provide strong evidence that prenatal exposure to both elevated endogenous maternal GC and synthetic glucocorticoids (sGC) alter fetal brain development and consequently impact upon behavior [6,10,11], including hyperactivity [12] and attention [13]. However, without experimental evidence in humans the effect cannot be confirmed. The routine administration of sGC in cases of threatened pre-term birth offers an opportunity to study whether prenatal exposure to GC is associated with long-term programming of behavior in humans in a quasi-experimental manner. 

sGC is commonly administered to pregnant women when pre-term birth is impending to accelerate fetal lung maturation and thereby reduce the risk of respiratory distress syndrome, and neonatal mortality [14]. Yet, very little is known about the long-term effects of prenatal sGC treatment on child behavior, including ADHD symptoms. The few existing studies report inconsistent findings. Some studies report an association between repeated prenatal sGC treatment and distractibility, hyperactivity and aggressive behavior [15], as well as attention problems [16] in young children, but others do not [17-19]. Generally, studies are limited by short follow-up times (young children only) and mostly examine the impact of repeated doses of prenatal sGC, and so little is known about the long-term impact of low/infrequent doses of prenatal sGC exposure on later child behavior. This is particularly important given that current guidelines recommend that only a single course of sGC should be administered (either 2 doses of 12mg of betamethasone or 4 doses of 6mg of dexamethasone) because of concerns regarding potential long-term effects of repeated sGC treatment [20]. One study examined the long-term association and reported that adults at age 31 who received a single course of prenatal sGC did not differ on mental health outcomes from those in the placebo condition [21]. However, the placebo group in this study received cortisone acetate with a 70^th^ of sGC potency, and so the impact of sGC from non-exposure cannot be completely assessed. Further studies are thus needed to examine this association. 

Besides the potential impact on the fetal brain, prenatal exposure to sGC treatment in humans has been linked with reduced birth size [22]. Small size at birth, in turn, has been implicated as a risk factor for child mental health [23]. It is possible that small birth size, which is a marker of suboptimal intrauterine conditions, may reflect altered brain development [23]. Prenatal exposure to maternal stress has also been linked to reduced birth size [24], with excess maternal GC as a potential causal mechanism [6]. The placenta, which normally acts as a barrier to regulate fetal exposure to endogenous maternal GC (inactivating excess cortisol to cortisone) [25], may play a key role in GC programming [26]. Prenatal exposure to sGC and maternal stress have also been associated with altered placental size [27,28], which in turn has been linked with child and adolescent mental health [29]. Changes in placental size can affect fetal nutrient and hormone supply [30], resulting in altered fetal growth and organ development, including the brain. Thus, it is possible that the GC-mental health link is mediated by deviation in either birth size and/or placental size. 

To clarify previous inconsistent findings, we examined data from a large, longitudinal cohort following children and adolescents. In studies examining prenatal sGC effects on child mental health, treatment-selection bias is a main issue, which we address here. It is essential to disentangle the potential effect of treatment from the conditions precipitating treatment. Our large dataset enabled us to very accurately match prenatally sGC exposed (cases) and unexposed (controls) children on baseline characteristics related to sGC treatment, by means of propensity-score-matching. However, an important limitation of propensity-score-matching is that, particularly in large studies, many unmatched controls are excluded from analysis, resulting in loss of data which may reduce the precision of the estimated association between the treatment and outcome [31]. Thus, we also used the entire cohort to analyse the data – matching each case to all possible controls on important confounders. The two matching procedures allowed us firstly to isolate the impact of prenatal sGC exposure on mental health from the confounding effects of treatment, and secondly, to examine the robustness of the results, thereby addressing important limitations of previous research. Further, we investigated whether birthweight and placental weight mediate the association between prenatal sGC treatment and offspring mental health to gain insight into the potential causal pathway. This is the first study to investigate the long-term impact of prenatal sGC treatment (low/infrequent doses) versus no treatment on mental health, particularly ADHD symptoms, in childhood (8 years) and again in adolescence (16 years). We hypothesise that prenatal sGC treatment will be related to poor mental health outcomes. 

## Materials and Methods

### Participants

The Northern Finland Birth Cohort (NFBC) 1986 recruited women in early pregnancy with an expected date of delivery between July 1, 1985 to June 30, 1986; 99% participated. Prospective data was gathered from pregnancy to child age 16 years. The cohort consists of 9479 births in Oulu and Lapland provinces. Here, we include N=8954 liveborn singletons with consent to use their data (exclusions: 226 twins, 6 triplets and 249 without consent). 

All pregnant women, literate in Finnish, were consecutively recruited at their first prenatal health care visit to tax-paid prenatal health services, which offer high-quality standardized care used by essentially all women in the country [32]. Women provided information via structured self-report questionnaires. Antenatal clinical and birth outcome data were obtained from maternity health centres and hospital medical records (completed by midwives during pregnancy and at birth), and abstracted onto study forms. As the original NFBC 1986 dataset did not include data on prenatal sGC treatment, we screened for potential sGC cases by performing a systematic chart review (see Figure S1). 

The cohort was followed-up at child ages 8 years (n=8106; 91% of original sample) and 16 years (n=6934; 77% of original sample), and focused on child health and well-being. Follow-ups were carried out using the national population-based registries, which identify all residents by unique personal numbers, to obtain current addresses. Thus, participants could be traced even outside the original geographic area.

The ethics committee of Northern Ostrobotnia Hospital District approved the study, and both parents and adolescents gave written informed consent. 

### Predictor: Prenatal sGC Treatment

In Finland in 1985/86, prenatal sGC treatment was administered in rare cases (at the discretion of the medical practitioner) as use of sGC during pregnancy was still controversial at the time, which explains the relatively few number of sGC cases in our study. There was no standard protocol for sGC treatment in the NFBC 1986 cohort, although caution was taken as only small and infrequent doses were administered. Dexamethasone (n=33) or betamethasone (n=4) - the drugs of choice for threatened pre-term birth, were administered. At 8 and 16 years, n=37 and n=29 cases respectively were available for analysis. Out of the 37 cases (at 8 years), 13 received a single sGC dose, 23 received 2 doses and the dose number for 1 case was not recorded. The total dosage ranged from 10mg to 25mg (the maximum total dosage equates approximately to a single course of sGC treatment as recommended by current guidelines). We obtained data on sGC treatment, number of sGC doses, total sGC dose and the interval between prenatal sGC exposure and birth (days), from medical records. 

Fetal exposure to GC is regulated by placental 11β-hydroxysteroid dehydrogenase type 2 (11β-HSD2) – an enzyme which normally inactivates 50-90% of endogenous maternal GC [25,33], but does not extensively metabolize sGC. Placental 11β-HSD2 inactivates only about 2% of dexamethasone and 7% of betamethasone [34], allowing the majority of sGC to cross the placenta to exert its intended therapeutic effect on fetal tissues. In contrast, prednisone (n=2) and hydrocortisone (n=1) have minimal placental transfer and so are typically administered to treat maternal medical conditions (e.g. allergic or inflammatory diseases) and were excluded from the analyses. 

Betamethasone and dexamethasone are long-acting substances (with biological half-lives ranging between 36 and 54 hours) [35], so it is unlikely that sGC treatment close to the time of birth could significantly impact fetal brain development as there would not be sufficient time for the drug to induce maximum effect. Thus, we excluded cases who had been exposed to sGC ≤4 days prior birth (n=11).

### Potential Mediators: Birthweight and Placental Weight

Birthweight (grams) was measured accurate to ±10g, immediately after birth by medical personnel. Placentas were washed with water and then weighed (including membranes and umbilical cord, cut approximately 5cm from the neonate) to the nearest gram within 30 minutes after birth, according to standard protocols [29].

### Outcome: Child and Adolescent Mental health

Teachers assessed child behavior at the age of 8 years using the Rutter B2 scale [36], a well-validated screener for childhood mental health. Each of the 26 items is rated as either it ‘certainly applies’ (scored 2), ‘applies somewhat’ (scored 1) or ‘does not apply’ (scored 0); yielding a total score between 0 to 52. The questionnaire generates three sub-scores: neurotic, antisocial and inattention-hyperactivity. Additionally, we examined the core ADHD symptoms individually i.e. inattention and hyperactivity. 

Parents reported adolescent behavior at 16 years using the Strengths and Weaknesses of ADHD symptoms and Normal behavior (SWAN) scale [37]. The SWAN consists of 18 items based on the symptoms of ADHD listed in the DSM-IV (9 items in the inattention subscale, 9 items in the hyperactive-impulsivity subscale, and together the 18 items indicate ADHD combined subtype). As this scale measures both weaknesses (scored 3, 2, 1) and strengths (scored -1, -2, -3), along with average behavior (scored 0), it is expected to produce a normal distribution of behavioral scores, thereby reducing the risk of over/under identifying ADHD behavior. 

Adolescents provided mental health self-reports at 16 years by completing the Youth Self-Report (YSR) [38] – a widely used questionnaire, derived from the Child Behavior Check List (CBCL), for use by 11-18-year-olds. The YSR includes 112 items covering behavioral and emotional problems, which are scored on a three-point scale (‘certainly applies’, ‘somewhat applies’ and ‘does not apply’, scored 2, 1 and 0, respectively). The YSR total problem score taps withdrawal, somatic complaints, anxiety/depression, thought problems, social problems, attention problems, delinquent behavior and aggressive behavior.

### Confounders

We considered potential confounders related to sGC treatment and child mental health which were available in the cohort. Socio-demographic factors previously associated with ADHD symptoms were: sex, maternal age (years), maternal education (either ≥11 years of education or <11 years of education, coded 0 or 1, respectively) and family structure (either married/co-habiting or single/widowed/divorced, coded 0 or 1, respectively) [39-41]; the latter three factors were measured at recruitment. Medical factors previously associated with child mental health or relevant for this study were: gestational age [42], total prenatal sGC dose (mg), interval between prenatal sGC exposure and birth (days), parity (continuous) [43], pre-pregnancy body mass index (BMI) (pre-pregnancy weight [kg] / height^2^ [m^2^]) (continuous) [32], and smoking during pregnancy (no/yes, coded 0/1, respectively) [40]. We obtained data on the main pregnancy complications related to pre-term birth from hospital records: gestational hypertension (no/yes), pre-eclampsia (no/yes) and placenta previa (no/yes). 

### Statistical Analysis

We used two analytical strategies to analyse the data: (1) analysis of the propensity-score-matched subsample by linear multiple regression, and (2) analysis of the entire sample by mixed-effects modeling. All main analyses were performed using SPSS 20.0, while the power analysis was run using G*Power 3 [44]. 

### Descriptive Analysis

We carried out descriptive analyses of all covariates potentially associated with sGC treatment, by means of t-test or chi-square statistics. Further, we examined whether there were any significant differences between matched cases and controls (within the propensity-score-matched subsample) by the covariates by means of t-test or chi-square statistics.

We performed attrition analyses at each follow-up to determine any differences in socio-demographics, birth outcomes and mental health outcomes between participants and non-participants.

### Matching Procedure

We used two matching procedures. First, we used propensity-score-matching [45] to match sGC cases and controls. The propensity score is the probability of treatment assignment based on observed baseline covariates. Matching on the propensity score creates balance, i.e. similarity, between cases and controls on the distribution of baseline covariates and thus reduces confounding associated with receipt of treatment. This matching technique mimics the randomization procedure prior to treatment allocation in a Randomized Controlled Trial (RCT). Thus, propensity-score-matching facilitates estimation of treatment effects using observational data. 

The covariates associated with sGC treatment were included as predictors in the logistic regression model used to calculate the propensity scores. The propensity scores were log transformed to normalize the distribution of the scores. sGC cases were matched to controls on the logit propensity score, using “nearest neighbour matching” with a caliper width (matching range) of ± 0.171402 (0.2 SD of the mean logit of the propensity score) [46]. We capitalized on our large dataset by matching each sGC case to 5 controls; ratio matching has been shown to be advantageous, and the optimum matching is normally reached with 5 matches to a single case [47]. This resulted in a sample of 222 children at 8 years (sGC cases, n=37; controls, n=185) and a sample of 174 adolescents at 16 years (sGC cases, n=29; controls, n=145). 

The second matching procedure took full advantage of the entire cohort by matching each sGC case, n=37, to all possible controls, n=6079, on gestational age and sex – confounders selected based on *a priori* information. Pre-term birth is a well-known risk factor for poor mental health outcomes [42] and is associated with gestational complications [48]. There is evidence that male fetuses are more vulnerable to prenatal insults [49], and are at an increased risk of psychiatric disturbance in childhood [41,50]. Thus, by matching on these known risks, we were able to isolate the impact of prenatal sGC exposure on mental health from the confounding effects of pre-term birth and sex. 

A “grouping” variable, based on gestational age and sex, was used to match the cases and controls. There were no sGC cases born within gestational weeks 41 to 43, therefore the 2229 controls born within those gestational ages could not be compared with the cases and consequently excluded from all subsequent analyses. At 8 years, 6116 children were available for analysis (sGC cases, n=37; controls, n=6079) and by 16 years of age, 5108 adolescents participated (sGC cases, n=29; controls, n=5079). This analytical strategy allowed us to use the greatest number of possible controls per case, thereby enhancing the precision of the analysis by maximizing use of all the available data [51]. 

### Regression Models

We used linear multiple regression to investigate the association between prenatal sGC treatment and child mental health, within the propensity-score-matched subsample. Prenatal sGC treatment was dichotomized: sGC case (coded 1) and sGC control (coded 0). The mental health scores were continuous. We adjusted for all potential confounders as shown by our descriptive analysis or by previous research. We used Cohen’s *f* ^2^ as an effect size estimator for the associations. 

We used mixed-effects modeling to re-analyse the association between prenatal sGC and mental health, but here we used the entire cohort. In this way, we can determine if the results are replicable or merely due to certain characteristics in the subsample. This statistical technique is robust in the analysis of unbalanced data, and thus is suitable here where there are unequal numbers of cases and controls. In the model, the predictor (prenatal sGC) and confounders were included as fixed effects. The “grouping” variable, based on gestational age and sex, was included as a random effect, thus allowing the model representing the impact of sGC on mental health to vary as a function of the group, thereby reducing the confounding effects of pre-term birth and sex. 

### Mediation Analysis

We used the bootstrap method [52,53] to evaluate whether birthweight and placental weight mediated the possible association between prenatal sGC and mental health, within the propensity-score-matched subsample. This is a resampling method which generates accurate confidence intervals to assess mediation effects. Bootstrapping does not impose any assumption about the shape of the distribution of the mediation effect, and thus it has been suggested that it is a more powerful technique than single sample methods [52,54]. 

### Power Analysis

We performed a *post hoc* power analysis to determine whether our study was sufficiently powered to detect any possible significant impact of sGC treatment, at 8 years and 16 years. 

## Results

### Descriptive Analysis

Table S1 shows pregnancy and birth characteristics for the unmatched cases and controls, available for analysis. Prior to propensity-score-matching, sGC cases and controls differed significantly on gestational age, birthweight, and placental weight. The difference on pre-pregnancy BMI was significant, p=.04 (based on all treated cases, n=41). As gestational age and pre-pregnancy BMI precede sGC treatment, these covariates were included in the propensity-score model. 

Table 1 shows pregnancy and birth characteristics for the propensity-score-matched cases and controls. There were no significant differences between the matched sGC cases and controls on any of the socio-demographic or medical factors. Importantly, there were no significant differences on gestational age and pre-pregnancy BMI, nor on the mean logit propensity score (case mean=-4.35; control mean=-4.36; p=.96) – indicating balance between cases and controls on treatment-associated confounders. 

**Table 1:** Pregnancy and birth characteristics for the sGC cases^***a***^ (n=37) and matched controls (n=185); 1:5 matching ratio, matched on logit of propensity score.

**Characteristic**	**Mean ± SD or n (%)**		
	**Case**	**Control**	**P**
**Pregnancy**			
Maternal age (years)	29.2 ± 5.0	27.6 ± 5.2	.10
Family structure			.37
Married/co-habiting	36 (97.3)	173 (93.5)	
Single/widowed/divorced	1 (2.7)	12 (6.5)	
Education (years)			.92
<11	10 (31.3)	54 (32.1)	
≥11	22 (68.8)	114 (67.9)	
Parity			.98
0	11 (29.7)	60 (32.4)	
1	16 (43.2)	76 (41.1)	
2	6 (16.2)	32 (17.3)	
≥ 3	4 (10.8)	17 (9.2)	
Smoking during pregnancy			.17
No	31 (86.1)	137 (75.7)	
Yes	5 (13.9)	44 (24.3)	
Pre-pregnancy BMI	21.1 ± 2.5	20.5 ± 2.6	.22
Pre-pregnancy BMI categories			.44
< 20	14 (37.8)	87 (47.0)	
20-24.99	20 (54.1)	90 (48.6)	
≥ 25	3 (8.1)	8 (4.3)	
Main pregnancy complications – risk for pre-term birth			
Gestational hypertension	0 (.0)	13 (7.1)	.23
Pre-eclampsia	1 (5.3)	4 (2.2)	.41
Placenta previa	0 (.0)	1 (.5)	.65
Total sGC dose (mg)	15.4 ± 4.6		
Interval between prenatal sGC exposure and birth (days)			
6-14	5 (13.5)		
15-23	0 (.0)		
24-32	6 (16.2)		
33-41	8 (21.6)		
41-49	10 (27.0)		
50-58	4 (10.8)		
59-67	2 (5.4)		
≥ 68	2 (5.4)		
**Birth**			
Sex			.86
Male	19 (51.4)	98 (53.0)	
Female	18 (48.6)	87 (47.0)	
Gestational age at birth (weeks)	37.2 ± 2.0	37.5 ± 2.0	.60
Gestational age categories (weeks)			.90
Pre-term birth (< 37)	10 (27.0)	48 (25.9)	
Term birth (≥ 37)	27 (73.0)	137 (74.1)	
Birthweight (g)	3159 ± 688	3151 ± 636	.95
Birthweight categories (g)			.59
< 2500	6 (16.2)	23 (12.4)	
2500-4499	30 (81.1)	160 (86.5)	
≥ 4500	1 (2.7)	2 (1.1)	
Placental weight (g)	586 ± 139	588 ± 135	.96
Placental weight categories (g)			.90
< 550	14 (37.8)	75 (40.8)	
550-719	17 (45.9)	84 (45.7)	
≥ 720	6 (16.2)	25 (13.6)	

*a* Including cases exposed to prenatal sGC > 4 days prior to birth.

Table S2 shows the attrition analyses among sGC cases from birth to 8 years, and from 8 years to 16 years. There were no significant differences by socio-demographics and birth outcomes between the participants and non-participants at 8 years. Similarly, attrition was not characterised by any significant differences from childhood to adolescence by socio-demographics, birth outcomes and mental health (at 8 years). 

While all the sGC cases were hospitalized, only one sGC case experienced one of the main pregnancy complications related to pre-term birth (gestational hypertension, pre-eclampsia or placenta previa). This single case did not significantly impact upon the mean mental health scores, and therefore was included in all analyses. Out of the controls, approximately 15% were hospitalized and 9% experienced the main pregnancy complications related to pre-term birth. 

### Regression Models

Table 2 shows the linear multiple regression results for the association between prenatal sGC treatment and mental health outcomes in children and adolescents, controlled for sex, birthweight, placental weight, socio-demographic factors (maternal age, education and family structure), and medical factors (total prenatal sGC dose, interval between prenatal sGC exposure and birth (days), parity and smoking during pregnancy). There were significant associations between prenatal sGC treatment and the total Rutter and inattention scores, at 8 years. The effect sizes for the total Rutter, inattention-hyperactivity, inattention and antisocial scores were moderate, while the association for the hyperactivity score showed a large effect size. Similar to the results at 8 years, we found consistent significant associations between prenatal sGC treatment and each of the outcome scores at 16 years; however, these did not reach statistical significance. 

**Table 2:** Linear multiple regression results for the association between prenatal glucocorticoid treatment (cases^***a***^, n=37 (at 8y) n=29 (at 16y), and controls balanced on gestational age and pre-pregnancy BMI, by means of logit of propensity score; 1:5 matching ratio) and mental health outcome scores for children and adolescents, adjusted for relevant confounders^***b***^.

**Mental Health**	**Prenatal glucocorticoid (GC) treatment (case/control)**								
	**Unadjusted**				**Adjusted^*b*^**				
	**B**	**95% CI for B**	**β**	**P**	**B**	**95% CI for B**	**β**	**P**	**Cohen’s *f*^2^**
**8-y-olds Rutter (teacher report)**									
Total Rutter score	1.07	-.95-3.10	.08	.30	8.34	.23-16.45	.56	.04	.23
Antisocial score	.28	-.45-1.00	.05	.45	2.93	-.04-5.9	.54	.05	.20
Neurotic score	.11	-.39-.62	.03	.66	1.55	-.55-3.67	.42	.15	.11
Inattention-hyperactivity score	.21	-.40-.72	.04	.58	2.16	-.03-4.35	.52	.05	.33
Inattention score**^*c*^**	-.02	-.23-.18	-.02	.81	.97	.16-1.80	.64	.02	.23
Hyperactivity score**^*d*^**	.19	-.21-.58	.07	.35	1.19	-.29-2.67	.41	.12	.36
**16-y-olds SWAN (parent report)**									
Combined ADHD score	-1.15	-8.48-6.19	-.03	.76	16.20	-14.65-47.04	.34	.30	.11
Inattention score**^*e*^**	-.53	-4.31-3.24	-.02	.78	9.00	-6.42-24.41	.37	.25	.11
Hyperactivity score**^*f*^**	-.61	-4.60-3.38	-.02	.76	7.20	-9.85-24.26	.27	.41	.10
**16-y-olds YSR (self-report)**									
YSR Total Problem score	2.10	-4.55-8.74	.05	.53	2.30	-26.43-31.00	.05	.88	.12

*a* Including cases exposed to prenatal sGC > 4 days prior to birth.

*b* adjusted for sex, birthweight, placental weight, socio-demographic factors (maternal age, education and family structure), and medical factors (total prenatal sGC dose, interval between prenatal sGC exposure and birth (days), smoking during pregnancy and parity).

*c* score based on Rutter item number 16 (range 0 to 2).

*d* score based on sum of Rutter items 1 and 3 (range 0 to 4).

*e* score based on sum of 9 SWAN items (range -27 to 27).

*f* score based on sum of 9 SWAN items (range -27 to 27).

Table 3 shows that the mixed-effects model produced very similar results to the first analysis. Prenatal sGC treatment was significantly associated with the total Rutter and inattention scores at 8 years, and was consistently associated with higher scores on all other outcomes at 8 and 16 years. Additionally, this method revealed neurotic scores were also elevated among sGC cases in comparison to controls at 8 years.

**Table 3:** Mixed-effects model for the association between prenatal glucocorticoid treatment (case^***a***^ vs. control, matched for gestational age and sex) and mental health outcome scores for children and adolescents, adjusted for relevant confounders^***b***^.

**Mental health**	**Prenatal glucocorticoid (GC) treatment (case/control)**							
	**Estimates**				**Pair-wise comparisons**			
	**n**	**Means**	**SE**	**95% CI**	**Mean difference (B)**	**SE**	**95% CI**	**P**
**8-y-olds Rutter (teacher report)**								
Total Rutter score					8.04	3.34	1.49-14.60	.02
*GC control*	6059	3.59	.32	2.90-4.28				
*GC case*	37	11.63	3.33	5.11-18.15				
Antisocial score					2.15	1.12	-.04-4.35	.05
*GC control*	6065	.75	.11	.50-1.00				
*GC case*	37	2.90	1.11	.72-5.01				
Neurotic score					2.48	.84	.84-4.12	.00
*GC control*	6076	.65	.02	.61-.70				
*GC case*	37	3.13	.83	1.50-4.76				
Inattention-hyperactivity score					1.53	1.01	-.43-3.51	.13
*GC control*	6069	3.86	.11	3.62-4.09				
*GC case*	37	5.39	1.00	3.43-7.36				
Inattention score**^*c*^**					.79	.35	.12-1.47	.02
*GC control*	6079	.22	.03	.16-.29				
*GC case*	37	1.01	.34	.34-1.69				
Hyperactivity score**^*d*^**					.74	.73	-.69-2.17	.31
*GC control*	6075	2.63	.08	2.46-2.81				
*GC case*	37	3.37	.73	1.95-4.80				
**16-y-olds SWAN (parent report)**								
Combined ADHD score					13.92	12.83	-11.22-39.06	.28
*GC control*	4950	-20.23	.86	-22.10- -18.36				
*GC case*	29	-6.31	12.77	-31.34-18.72				
Inattention score**^*e*^**					8.42	6.80	-4.91-21.74	.22
*GC control*	4950	-8.00	.49	-9.05- -6.94				
*GC case*	29	.42	6.77	-12.85-13.69				
Hyperactivity score**^*f*^**					5.47	6.99	-8.24-19.17	.43
*GC control*	4950	-12.22	.38	-13.05- -11.38				
*GC case*	29	-6.75	6.96	-20.39-6.89				
**16-y-olds YSR (self-report)**								
YSR Total Problem score					16.39	12.05	-7.24-40.02	.17
*GC control*	5079	25.52	1.34	22.67-28.36				
*GC case*	29	41.91	12.03	18.33-65.49				

*a* Including cases exposed to prenatal sGC > 4 days prior to birth.

*b* adjusted for birthweight, placental weight, socio-demographic factors (maternal age, education and family structure), and medical factors (total prenatal sGC dose, interval between prenatal sGC exposure and birth (days), smoking during pregnancy, parity and pre-pregnancy BMI).

*c* score based on Rutter item number 16 (range 0 to 2).

*d* score based on sum of Rutter items 1 and 3 (range 0 to 4).

*e* score based on sum of 9 SWAN items (range -27 to 27).

*f* score based on sum of 9 SWAN items (range -27 to 27).

### Mediation Analysis

The bootstrap method showed that there were no significant indirect effects of birthweight (e.g. for total Rutter score, bootstrap estimate=.67, percentile 95% CI=-1.33-3.00) or placental weight (e.g. for total Rutter score, bootstrap estimate=.24, percentile 95% CI=-1.07-2.00) on the sGC-mental health pathway. Thus, we did not find evidence for mediation by birthweight or placental weight.

### Power Analysis

The *post hoc* power analysis showed that the study had sufficient power to detect significant differences at 8 years (e.g. for total Rutter score model, 1-β=.80, with an effect size *f* ^2^=.23 and p=.05), but was under-powered at 16 years (e.g. for combined ADHD score model, 1-β=.39, with an effect size *f* ^2^=.11 and p=.05).

## Discussion

This study is the first to explore the long-term associations between prenatal exposure to sGC treatment and mental health in childhood and adolescence. We found that both children and adolescents prenatally exposed to sGC scored consistently higher on internationally validated screening instruments of mental health, by teacher, parental and self-reports, than controls. The propensity-score-matched subsample showed that prenatal exposure to sGC treatment was significantly associated with the total Rutter and inattention scores in childhood, independent of relevant confounders – sex, birthweight, placental weight, socio-demographic factors and medical factors. Past studies have in particular found it challenging to disentangle the effect of prenatal sGC on mental health from pre-term birth, which is associated with both sGC treatment and child mental health. Through propensity-score-matching, cases and controls were balanced, i.e. matched, on gestational age and pre-pregnancy BMI, and so we were able to isolate the impact of prenatal sGC on mental health from these treatment-associated confounders. Therefore, our findings suggest that prenatal sGC is a potential programming agent of child mental health, rather than a mere epiphenomenon. We examined the robustness of our findings by testing the association using the entire cohort by means of mixed-effects modeling. We found very similar results using the entire sample compared with the subsample, providing further evidence that our results are unlikely to be affected by confounding.

We set out to examine the potential long-term association between prenatal sGC exposure and mental health, and therefore studied adolescents by way of parental-report specific for ADHD symptoms and self-report for general mental health. Given attrition by the 16-year follow-up, only 29 cases remained for analysis which left our study under-powered at this point - as confirmed by our power analysis. Nonetheless, the pattern of associations at 16 years was consistent with the findings reported at 8 years. 

Our findings corroborate and extend previous results of observational studies of high-risk pregnancies in humans, which have examined the effect of repeat prenatal sGC doses on child mental health [15,16]. Most previous studies have used high-risk samples in comparison to normal pregnancies, making it difficult to differentiate between medical complications that prompted treatment and the potential effect of the treatment itself on the outcome. Ours is a community cohort in which both controls and cases experienced pregnancy complications and were hospitalized. Out of the controls, approximately 9% experienced the most common causes of pre-term birth (gestational hypertension, pre-eclampsia and placenta previa) and 15% were hospitalized. Only one case experienced one of the most common causes of pre-term birth. In this sense, our results are more generalizable, rather than specific to high-risk sub-groups and so are unlikely to be affected by confounding of pregnancy complications.

We were able to examine the impact of fairly low and infrequent doses of prenatal sGC (the average total dose was 15.4mg and the maximum dosage was approximately equal to a single course of sGC, according to current guidelines). Given the concerns raised by use of repeat sGC in pregnancy [14,22] and the call for longitudinal research [55], it is of public health interest to study long-term risks associated with exposure at this low dosage. Our findings suggest that even at low dosages the fetal brain may be sensitive to sGC. Interestingly, we found that prenatal sGC had a non-specific effect on child mental health, as indicated by an association with the total Rutter, which reflects a range of emotional and behavioral problems, including ADHD symptoms. A total Rutter score of ≥9 indicates probable psychiatric disturbance, and so the mean Rutter score difference of approximately 8 points between cases and controls reflects clinical significance. 

Cortisol may directly impact brain development because glucocorticoid receptors (GR) and mineralocorticoid receptors (MR), both of which have a high affinity for GC, are highly expressed in the fetal brain [56], particularly the hippocampus [57]. Animal studies have shown that prenatal sGC exert widespread effects on the developing brain, reducing neuron proliferation [58], as well as affecting neuron structure and synapse formation [59]. Prenatal sGC has been linked with reduced density of hippocampal neurons in the offspring, in both humans and animals [57,60]. Altered hippocampal structure in turn has been associated with mental health, including ADHD [61,62]. A recent study demonstrated that prenatal sGC was associated with thinner brain cortex in children, which in turn was linked with affective problems [63]. There is also evidence that prenatal sGC has a long-term impact on hypothalamic-pituitary-adrenal (HPA) axis reactivity in term-born children, which may bear significant implications for stress-related psychiatric disorders [64]. 

We tested the hypothesis that deviation in birthweight or placental weight would mediate the association between prenatal exposure to sGC and child mental health. It is possible that altered birth size and/or placental size, both of which have been linked to prenatal sGC exposure [22,27] and child mental health [23,29], would lie on the GC programming pathway. However, we did not find support for this idea.

As in all longitudinal studies, attrition occurs at every follow-up and is the main limitation. With a loss of 9 cases by the 16-year follow-up, our study was under-powered at this point, which is a likely explanation for non-significant findings at this age. The NFBC 1986 is a prospective cohort but was not designed to examine sGC treatment outcomes - we performed a chart review to identify sGC cases, thus we cannot rule out the impact of unmeasured confounders. It is not possible to completely rule out that the observed differences in mental health scores may be due to the complications of pregnancy which prompted sGC treatment. However, this seems unlikely here as both cases and controls experienced pregnancy complications, and our matching procedures ensured that cases and controls were balanced on important confounders. Due to the very small number of sGC cases experiencing pregnancy complications known to be a risk for pre-term birth (n=1), we could not study these as sub-groups. Further work is required to determine the impact of pregnancy complications on later mental health with larger samples. Finally, sGC cannot be directly equated to endogenous maternal GC. sGC and endogenous GC largely bind to different types of steroid receptors and so may have different biological effects. Despite these limitations, sGC provides a useful quasi-experimental model in the absence of direct experimental manipulation in humans and provides a tentative proof of concept, warranting further research to better understand the associations and their underlying mechanisms.

Our study has important strengths. First, we used propensity-score-matching to account for treatment-selection bias (thereby partly mimicking an RCT), in particular gestational age and pre-pregnancy BMI, and so we were able to isolate the effect of the drug from these two significant confounders associated with receipt of treatment. Our large dataset enabled us to very precisely match cases to controls on the logit propensity scores. Thus the results presented here are not due to pre-maturity, which its threat would prompt treatment, and is known to be a risk for poor neurodevelopmental outcomes, including ADHD [65,66] nor pre-pregnancy BMI which was also associated with treatment in this sample as well as ADHD [32,67]. The matched cases and controls were also balanced on other important confounders, and these confounders were additionally adjusted for in the main analysis. Thus, we minimized confounding related to sGC treatment and mental health as much as possible. Second, we were able to replicate the results produced from the propensity-score-matched subsample using the entire cohort by means of mixed-effects modeling, demonstrating the robustness of our findings. Third, we used precise case classification (exposed >4 days prior to delivery) to ensure that the drug had sufficient time to act on the fetal brain. Studies which do not take exposure time into consideration e.g. Dalziel et al. (2005) may be more likely to report null findings as the drug may not have had time to act on the fetal brain. Fourth, we assessed child and adolescent mental health via multiple informants and multiple validated instruments, which strengthen the credibility of the results and extend previous findings that have relied almost completely on parental report. Fifth, we address the growing public health concern regarding side-effects of sGC treatment by studying the impact of fairly low/infrequent doses of sGC. 

In conclusion, the data we present here, originating from a population-based cohort, is the largest to date and show an association between prenatal sGC exposure and child mental health. Further work is necessary to confirm the long-term associations. By capitalizing on the natural experiment in which women are treated with sGC, we were able to explore the hypothesized pathway between fetal glucocorticoid exposure and later child mental health. The results show that this pathway merits further scientific research, though it is a challenge using human studies. While the benefits of prenatal sGC treatment on the immediate health and survival of the pre-term neonate are clear, it is also important to consider the long-term health implications of this drug, including those relating to mental health. The clinical ramifications of this study call for close monitoring of children prenatally exposed to sGC in order to provide support early if mental health problems arise. 

## Supporting Information

Figure S1 **Flowchart of systematic screening process to identify sGC cases within the NFBC 1986.**
(TIF)

Table S1 **Pregnancy and birth characteristics for the sGC cases^a^ (n=37) and controls (n=8018), available for analysis.**
(DOCX)

Table S2 **Attrition analyses from birth to 8 years and 8 to 16 years, among sGC cases^a^.**
(DOCX)
