# Supplementary material for: Prenatal Glucocorticoid Treatment and Later Mental Health in Children and Adolescents
Source: PLoS One. 2013 Nov 22;8(11):e81394. doi: 10.1371/journal.pone.0081394 (PMC3838350; doi:10.1371/journal.pone.0081394)
Supplement: Table S1 — Pregnancy and birth characteristics for the sGC casesa (n=37) and controls (n=8018), available for analysis. (DOCX) [file pone.0081394.s002.docx]

| **Table S1:** Pregnancy and birth characteristics for the sGC cases^a^ (n=37) and controls (n=8018), available for analysis. | | | |
| --- | --- | --- | --- |
| **Characteristic** | **Mean ± SD or n (%)** | |  |
|  | **Case** | **Control** | **P** |
| **Pregnancy** |  |  |  |
| Maternal age (years) | 29.2 ± 5.0 | 28.2 ± 5.5 | .26 |
| Family structure |  |  | .53 |
| Married/co-habiting | 36 (97.3) | 7606 (95.1) |  |
| Single/widowed/divorced | 1 (2.7) | 393 (4.9) |  |
| Education (years) |  |  | .90 |
| <11 | 10 (31.3) | 2186 (30.2) |  |
| ≥11 | 22 (68.8) | 5044 (69.8) |  |
| Parity |  |  | .63 |
| 0 | 11 (29.7) | 2713 (34.0) |  |
| 1 | 16 (43.2) | 2658 (33.3) |  |
| 2 | 6 (16.2) | 1439 (18.0) |  |
| ≥ 3 | 4 (10.8) | 1178 (14.7) |  |
| Smoking during pregnancy |  |  | .39 |
| No | 31 (86.1) | 6263 (80.4) |  |
| Yes | 5 (13.9) | 1531 (19.6) |  |
| Pre-pregnancy BMI | 21.1 ± 2.5 | 22.4 ± 3.5 | .07 |
| Pre-pregnancy BMI categories |  |  | .10 |
| < 20 | 14 (37.8) | 1916 (24.5) |  |
| 20-24.99 | 20 (54.1) | 4557 (58.3) |  |
| ≥ 25 | 3 (8.1) | 1350 (17.3) |  |
| Main pregnancy complications – risk for pre-term birth |  |  |  |
| Gestational hypertension | 0 (.0) | 414 (5.2) | .15 |
| Pre-eclampsia | 1 (5.3) | 162 (2.0) | .78 |
| Placenta previa | 0 (.0) | 13 (.2) | .81 |
| **Birth** |  |  |  |
| Sex |  |  | .78 |
| Male | 19 (51.4) | 4083 (50.9) |  |
| Female | 18 (48.6) | 3935 (49.1) |  |
| Gestational age at birth (weeks) | 37.2 ± 2.0 | 39.5 ± 1.5 | .00 |
| Gestational age categories (weeks) |  |  | .00 |
| Pre-term birth (< 37) | 10 (27.0) | 283 (3.5) |  |
| Term birth (≥ 37) | 27 (73.0) | 7733 (96.5) |  |
| Birthweight (g) | 3159 ± 688 | 3591 ± 514 | .00 |
| Birthweight categories (g) |  |  | .00 |
| < 2500 | 6 (16.2) | 184 (2.3) |  |
| 2500-4499 | 30 (81.1) | 7548 (94.1) |  |
| ≥ 4500 | 1 (2.7) | 286 (3.6) |  |
| Placental weight (g) | 586 ± 139 | 648 ± 129 | .00 |
| Placental weight categories (g) |  |  | .02 |
| < 550 | 14 (37.8) | 1564 (19.6) |  |
| 550-719 | 17 (45.9) | 4292 (53.7) |  |
| ≥ 720 | 6 (16.2) | 2143 (26.8) |  |
| ^a^Including cases exposed to prenatal sGC > 4 days prior to birth. | | | |
