# Supplementary material for: Prenatal Glucocorticoid Treatment and Later Mental Health in Children and Adolescents
Source: PLoS One. 2013 Nov 22;8(11):e81394. doi: 10.1371/journal.pone.0081394 (PMC3838350; doi:10.1371/journal.pone.0081394)
Supplement: Table S2 — Attrition analyses from birth to 8 years and 8 to 16 years, among sGC casesa. (DOCX) [file pone.0081394.s003.docx]

| **Table S2:** Attrition analyses from birth to 8 years and 8 to 16 years, among sGC cases^a^. | | | | | | | |
| --- | --- | --- | --- | --- | --- | --- | --- |
|  | **Mean ± SD and n (%)** | | | | | | |
|  | **All at birth**  **(n=41)** | **Dropout (8y)**  **(n=4)** | **Remain (8y)**  **(n=37)** | **P** | **Dropout (16y) (n=9)** | **Remain (16y) (n=29^b^)** | **P** |
| **Socio-demographic factors (at birth)** |  |  |  |  |  |  |  |
| Family structure |  |  |  | .74 |  |  | .07 |
| Married/co-habiting | 40 (97.6) | 4 (100.0) | 36 (97.3) |  | 8 (88.9) | 29 (100.0) |  |
| Single/widowed/divorced | 1 (2.4) | 0 (.0) | 1 (2.7) |  | 1 (11.1) | 0 (.0) |  |
| Education |  |  |  | .35 |  |  | .08 |
| <11 years | 10 (29.4) | 0 (.0) | 10 (31.3) |  | 4 (57.1) | 6 (23.1) |  |
| ≥11 years | 24 (70.6) | 2 (100.0) | 22 (68.8) |  | 3 (42.9) | 20 (76.9) |  |
| **Birth outcomes** |  |  |  |  |  |  |  |
| Sex |  |  |  | .96 |  |  | .25 |
| Male | 21 (51.2) | 2 (50.0) | 19 (51.4) |  | 6 (66.7) | 13 (44.8) |  |
| Female | 20 (48.8) | 2 (50.0) | 18 (48.6) |  | 3 (33.3) | 16 (55.2) |  |
| Birthweight (g) | 3101 ± 738 | 2565 ± 1075 | 3159 ± 688 | .12 | 3023 ± 798 | 3128 ± 761 | .72 |
| Placental weight (g) | 597 ± 202 | 690 ± 551 | 586 ± 139 | .34 | 567 ± 159 | 583 ± 142 | .78 |
| **Mental health (8-y-olds)** |  |  |  |  |  |  |  |
| Total Rutter score |  |  | 5.2 ± 6.4 |  | 6.2 ± 7.8 | 4.9 ± 6.0 | .60 |
| Antisocial score |  |  | 1.2 ± 2.2 |  | 1.4 ± 2.8 | 1.0 ± 2.0 | .64 |
| Neurotic score |  |  | .9 ± 1.3 |  | 1.4 ± 1.3 | .8 ± 1.3 | .23 |
| Inattention-hyperactivity score |  |  | 4.1 ± 1.4 |  | 4.2 ± 1.6 | 4.0 ± 1.4 | .65 |
| Inattention score |  |  | .3 ± .5 |  | .2 ± .4 | .3 ± .5 | .89 |
| Hyperactivity score |  |  | 2.8 ± 1.1 |  | 3.0 ± 1.4 | 2.7 ± 1.0 | .50 |
| ^a^Including cases exposed to prenatal sGC > 4 days prior to birth. | | | | | | | |
| ^b^At 16 years, there was one extra participant who did not take part in the 8 year follow-up. | | | | | | | |
